# Coarse-to-Fine Satellite Images Change Detection Framework via Boundary-Aware Attentive Network

**DOI:** 10.3390/s20236735

**Published:** 2020-11-25

**Authors:** Yi Zhang, Shizhou Zhang, Ying Li, Yanning Zhang

**Affiliations:** 1 School of Computer Science, National Engineering Laboratory for Integrated Aero-Space-Ground-Ocean Big Data Application Technology, Shaanxi Provincial Key Laboratory of Speech & Image Information Processing, Northwestern Polytechnical University, Xi’an 710129, China; lybyp@nwpu.edu.cn (Y.L.); ynzhang@nwpu.edu.cn (Y.Z.); 2 School of Communication and Information Engineering, Xi’an University of Posts and Telecommunications, Xi’an 710121, China

**Keywords:** change detection, deep learning, attentive, coarse-to-fine, encoder-decoder architecture, end-to-end

## Abstract

Timely and accurate change detection on satellite images by using computer vision techniques has been attracting lots of research efforts in recent years. Existing approaches based on deep learning frameworks have achieved good performance for the task of change detection on satellite images. However, under the scenario of disjoint changed areas in various shapes on land surface, existing methods still have shortcomings in detecting all changed areas correctly and representing the changed areas boundary. To deal with these problems, we design a coarse-to-fine detection framework via a boundary-aware attentive network with a hybrid loss to detect the change in high resolution satellite images. Specifically, we first perform an attention guided encoder-decoder subnet to obtain the coarse change map of the bi-temporal image pairs, and then apply residual learning to obtain the refined change map. We also propose a hybrid loss to provide the supervision from pixel, patch, and map levels. Comprehensive experiments are conducted on two benchmark datasets: LEBEDEV and SZTAKI to verify the effectiveness of the proposed method and the experimental results show that our model achieves state-of-the-art performance.

## 1. Introduction

Timely and accurate change detection on bi-temporal satellite images that are taken at different times in the unique geographic location, is a task with practical application significance. Basically, the change in the bi-temporal image-pairs can be defined as the differences among attributes, positions, ranges etc. of the objects on the land surface. Recently, research on change detection has been an active topic with the rapid development of computer vision techniques [1,2,3,4,5].

There are various widely adopted methods proposed by researchers, covering variable detecting strategies and achieving grateful performances on a range of datasets. Traditional methods extracted low-level manual features and made decisions based on thresholds or clustering analysis. Traditional change detection methods can be roughly divided into two strategies: pixel-based and object-based [6]. The pixel-based methods [7,8] mainly set thresholds or cluster on the comparing results from the corresponding pixel-values directly. Generally, these simple methods often lead to certain “salt and pepper” noise, because of their ignorance on context information [2]. To alleviate the noise, several improved probabilistic models [9,10,11,12,13] are proposed. Pixel-based methods achieve far from satisfactory results, especially when used in the scenario of very high resolution (VHR) images [2]. Object-based approaches [14,15,16] emerge accordingly mainly following this pipeline: firstly, images are divided into targets, and then their bi-temporal relativeness is compared and analyzed. These targets contain rich information on spectral, textual, structural and geometric, providing support for subsequent similarity analysis.

More recently, deeply learned features with rich semantic information are introduced to replace the low-level manually designed features. Similar to the pipelines of traditional pixel-based methods, some of them take the deep neural networks as a robust feature extractor, instead of human designed descriptor which require a lot of domain knowledge, followed by various decision making strategies on those feature maps. The pre-trained Convolutional Neural Networks (CNNs) on natural image datasets, such as VGGNet and ResNet [17,18,19], are well-performed in remote sensing tasks [20]. In order to further adapt to the specific domain, Zhang et al. [21] trains a deep brief network (DBN) which extracts feature maps from raw data, and then conducts cluster analysis on them. To keep the respective field and feature dimensions, Zhan et al. [22] trains a Siamese network with AlexNet cutting off pooling layers to represent two images and then generate change map on the difference of them with contrastive loss. To enhance interclass discrimination and intraclass compactness, Zhang et al. [23] trains a Siamese network with an improved triplet loss. Different from extracting deep features from raw images, several approaches dealt with patches or superpixels from raw images, and then fed them into the deep learning models to learn the association between these graphs or sub-regions. By generating superpixels before learning, several approaches segment superpixels firstly, and then fed them into zoom out CNN [24], ResNet [25], stacked contrastive AutoEncoder [11] and Sparse De-noising AutoEncoder (SDAE) [26] and to generate multi-scale deep features. The Siamese CNN structure, which provides effective feature representation ability, is also widely used in multimodal image patch based methods, including optical images [27] and incomplete satellite images [28].

In addition, to make the change detection task end-to-end learnable, the fully convolutional network (FCN) related methods are proposed to make full use of the contextual information. For example, the typical FCN [29] tackles this problem by utilizing the context information and results in a better localization, through involving the combine feature maps at multiscale convolutional layers. Basically, there are two types of styles according to the input pattern: 2-stream and 2-channel [30]. The 2-stream means that two streams of networks share structure and weights and process images respectively. It is one of the most common used strategies in image classification or other related vision tasks. While 2-channel is to concatenate two images as one input of the network. It provides more flexibility and converges faster [30]. As for performance, 2-channel methods typically perform well on global features under the same convolutional backbones, but it is not the same effective when the pairs involve more local high-level features [31], while 2-stream methods can focus more on local information. Based on 2-stream architecture, Lei et al. [32] and Liu et al. [33] applies standard U-Net [34], as an image-to-image architecture, on bi-temporal images change detection task. Caye Daudt et al. [3] designed skip connections cross bi-temporal images on Siamese models, named FC-Siam-diff and FC-Siam-conc. Based on these baselines, the PGA-SiamNet [4] introduces a co-attention guide onto the bridge before decoding, to emphasize the importance of correlation among the feature maps pairs. In Zhang et al. [1], a deep supervised fusion strategy is introduced to the FC-Siam-conc like model, which is completed by using attention modules after on concatenated encoding feature maps and reconstructed maps, to suppress the irrelevant samples and spatial transformed refine the maps. By doing that, it improves the change maps boundary completeness and internal compactness. Instead of processing bi-temporal images separately, 2-channel architectures stack an image pair as one input to the networks. This strategy is applied in Alcantarilla et al. [35] for street view change detection. FC-EF in Caye Daudt et al. [3] stacks bi-temporal three channels RGB satellite images as an image with six channels feeding into a U-Net like model. Peng et al. [2] proposed to train a Nested U-Net (UNet++) [36] on VHR satellite images, that a more powerful U-Net like deep supervised model armed with dense skip connections, with applying a multiple fusion loss. This network can automatically explore and learn from low level to high level benefited by the dense structure and generate finer map by the deep supervised fusion loss.

However, there are still some disadvantages in existing methods. In terms of object-level change detection, especially under the scenario where disjoint changed areas that show variability in shapes are included in one change map, most of the existing methods are able to detect the changed areas with a promising recall rate. Furthermore, the boundaries of those areas are not detected accurately. To tackle the above problems, we design a coarse-to-fine change detection framework to process satellite image pairs with a boundary-aware attentive network (BA^2^Net), which utilizes the image-pairwise semantic information with an attentional strategy to locate multiple changed areas correctly and represent the boundaries of the changed areas with the refined encoder-decoder architecture progressively. Furthermore, we train the proposed BA^2^Net with a hybrid loss function from pixel, patch, and map levels. Experimental results on two public benchmark datasets LEBEDEV [37] and SZTAKI [9] show that the proposed model achieves state-of-the-art performance.

In summary, our main contribution can be summarized as follows: (1) We propose a BA^2^Net, which utilizes the image-pairwise semantic information with an attentional strategy to locate multiple small objects correctly and represent the boundaries of the changing area with the refined encoder-decoder architecture progressively. (2) A hybrid loss function which introduces supervisory signals from pixel, patch, and map levels to train the proposed BA^2^Net model. (3) We conduct comprehensive experiments and analysis on two public benchmark datasets LEBEDEV and SZTAKI and achieve state-of-the-art performances.

The remainder of the paper is organized as follows: Section 2 elaborates the proposed model and the training method. Section 3 shows the experimental results and ablation studies. Section 4 discusses the proposed framework. In Section 5, we draw the conclusion of the paper.

## 2. Methodology

**Figure 1:**
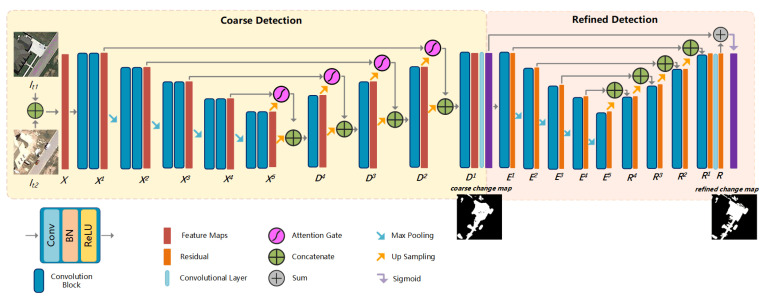
Illustration of our BA^2^Net architecture. The whole network contains two Encoding-Decoding subnets, each of which is composed of several convolutional blocks and skip connections. All convolutional blocks are with unified structures as *Convolutional + BatchNormalize + ReLU* layers with different parameters settings. The output of BA^2^Net, refined change map (RCM), is sigmoid sum of the coarse change map (CCM) and the refined residual (*R*).

In this section, we elaborate the proposed method and the training process. As shown in Figure 1, our model mainly consists of two subnets: coarse detection and refined detection. The coarse subnet is an encoder-decoder network with attention mechanism to generate the coarse change maps of the input image pairs. The refined subnet is a U-Net like network to refine the coarse detected map to final change map by adding the coarse detected map and the residual.

### 2.1. Coarse Detection

For change detection tasks, it is to input a bi-temporal image pair and then output a change map(CM) with original width and height size. This change map marks the changed and unchanged regions by labeling each pixel in it, which is to solve detection mapping function d(·) as follows,

(1) $$CM=d(I_{t1},I_{t2})$$

where CM means the change map, $I_{t1}$ and $I_{t2}$ mean the bi-temporal images respectively.

Among several straightforward solutions to this function d(·), it is the FCN frameworks to apply deep learning firstly, converting change detection task into a binary classification task on each pixels in CM under the fully supervision ground truths, which are the true results of the change maps provided by datasets which are typically labeled by human annotators. Based on the FCN framework, CD-Net [35] takes up-sampling and convolutions on the feature maps generated by continuous convolutions and down-samplings. To avoid the obvious information losing and errors accumulation, following the encoder-decoder framework of FCN, U-Net applies skip connections on each stage instead of directly supervising and making loss back propagation focusing on the high-level features. The stage in Section 2 refers to a bunch of convolutional blocks fed by the features on the same scale [34,38]. With the network going deeper, the stage ID goes higher. These skip connections ensure that the obtained features can consider sufficient information provided by low and high-level features. In standard U-Net, the skip connection is completed by concatenating the (n−1)-th maps with the up-sampled *n*-th maps to combine feature maps crossing blocks, where *n* refers to the stage ID and the update rule for the (n−1)-th block with the parameters $\Theta^{n-1}$ can be presented as follows,

(2) $$\frac{\partial (x_{i}^{n})}{\partial (\Theta^{n-1})}=\frac{\partial (f\left(x_{i}^{n-1};\Theta^{n-1}\right))}{\partial (\Theta^{n-1})}$$

However, U-Net based methods still maintain few weaknesses in false detection, especially when there are multiple changed regions in one image-pair. In the encoding phase, with the networks going deeper and consecutive stacking convolutional and pooling layers, multiscale features are generated. Shallow high resolution features are mapped to more image details, such as textures, lines and so on. Deep low resolution features can capture context information and highlight the category and location of foreground objects. In the change detection task, superior detection results are reflected in two aspects: one is to accurately detect the changed areas/objects, eliminating some semantic interference such as seasonal appearance differences; the other is to mark the changed area clearly, especially when there are a variety of object shapes and complex boundaries. These two points correspond to deep features and shallow features, respectively. Though U-Net involves multiple features by skip connections, its strategy of paying even attention to each stage may lead to the weaknesses described above.

**Figure 2:**
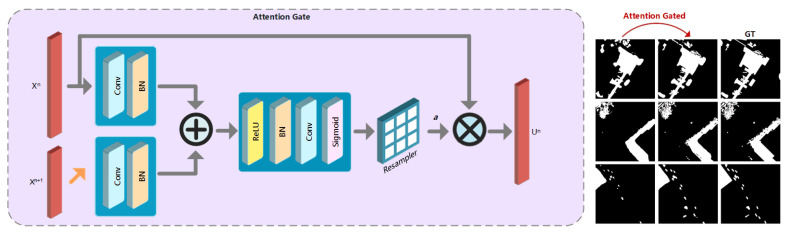
Illustration of attention gate (AG) architecture. The inputs to AG are feature maps from different stages, $X^{n+1}$ and $X^{n}$. The output of is $U^{n}=X^{n}\cdot a$, where *a* is the attention coefficients and · refers to element-wise multiplication. The attention gated change maps are closer to the ground truths (GTs).

Hence, we introduce the attention gate (AG) into our coarse detection. Benefiting from this soft attention mechanism, the network can be trained in more effectiveness. We introduce AGs to the change detection task by adding AGs into the skip connections of U-Net. The feature maps generated by deeper layers $X^{n+1}$ are used as the gating to guide eliminating the irrelevance caused by unchanged area or noise information. In the forward pass, AGs are performed right before the concatenation, and skip connections merge only the relevant neuron activations filtered by AGs. As shown in Figure 2, the $X^{n}$ and up-sampled $X^{n+1}$ are fed into two sets of ‘Convolutional+BN’ layer, independently.

Then the results are added as an input to ‘ReLU + BN + Convolutional + Sigmoid’ layers to learn the attention coefficients $a_{i}\in \left[0,1\right]$. Then the output of AG is $u_{i}^{n}=x_{i}^{n}\cdot a_{i}$. While the update rule for encoding convolutional functions f(·) with parameters Θ in block n−1 can be formulated as follows,

(3) $$\frac{\partial (u_{i}^{n})}{\partial (\Theta^{n-1})}=a_{i}^{n}\frac{\partial (f\left(x_{i}^{n-1};\Theta^{n-1}\right))}{\partial (\Theta^{n-1})}+\frac{\partial (a_{i}^{n})}{\partial (\Theta^{n-1})}x_{i}^{n}$$

It can be observed that, compared with Equation (2), the first gradient term in right side is scales with $a_{i}\in \left[0,1\right]$. The weight of the features obtained by shallower layers is reduced. At the same time, the features from deeper layers are more involved in the gradient updating. This design allows the network guided by deeper features which can capture context information and reduce the gradients of the weights from the unchanged pixels.

As shown in Figure 1, the coarse detection consists of several convolutional blocks, connecting by pooling operations or skip connections. Each block contains a Convolutional layer, a batch normalize (BN) layer and a rectified linear unit (ReLU) layer. Image pairs $Image_{t1}:\left[256\times 256\times 3\right],Image_{t2}:\left[256\times 256\times 3\right]$ are concatenated as one input X:[256×256×6] to the network. It firstly encodes input *X* by sets of convolutional blocks, that is $\left\{X\to X^{1}\to X^{2}\to X^{3}\to X^{4}\to X^{5}\right\}$, with the output feature map channel number $C_{p}^{n}$ of the *n*-th convolutional blocks, $X^{n}$ and $D^{n}$ as $C_{p}^{n}=\left\{64,128,256,512,1024\right\},n\in \left[1,5\right]$. The scale of $X^{n}$ is gradually reduced by pooling operation with the increase of stage ID *n*. In the decoding phase, feature maps are broadcasting along with $\left\{X^{5}\to D^{4}\to D^{3}\to D^{2}\to D^{1}\right\}$, each $D^{n}$ is the convoluted concatenation from two paths of information, $X^{n+1}$ after up-sampled and $X^{n}$ transmitted by the skip connection. The size of convolution kernel of all convolutional layers is set to 3×3, and all MaxPooling layers kernel sizes and strides are set as [2,2], each UpSampling layer scale factor equals 2. A Convolutional layer is applied on $D^{1}$ to obtain the single channel coarse map. In standard U-Net, the skip connection is completed by concatenating the *n*-th maps with the up-sampled (n+1)-th maps to combine feature maps crossing blocks. In our coarse detection, we introduce an attention mechanism into each connection.

### 2.2. Refined Detection

In general, compared with ground truths (GTs), the change maps obtained by coarse detection subnet are usually with fuzzy and noisy boundaries or unbalanced regional prediction probability [39], which are defined as coarse change map (CCM). We use residual refined detection to measure the difference between CCMs and GTs. The function of generating refined change map (RCM) is defined as:

(4) RCM=CCM+R

where *R* is the residual between CCM and GT. As shown in Figure 1, *R* is learned by the refined detection. To refine both region and the boundary, the refined detection is also a standard encoder-decoder structure supervised by GTs, which is constructed by convolutional blocks and standard skip connections. It has fewer blocks and channels than the previous coarse detection, with all $C_{r}^{n}=64,n\in \left[1,5\right]$.

### 2.3. Hybrid Loss

We adopt a hybrid loss function for the training of our proposed network, which is calculated as:

(5) $$L_{hybrid}=\lambda_{f}L_{focal}+\lambda_{t}L_{tversky}+\lambda_{s}L_{ssim}$$

where $L_{focal}$, $L_{tversky}$, $L_{ssim}$ denote focal loss [40], Tversky loss [41] and Structural SIMilarity (SSIM) loss [42], respectively, with $\left[\lambda_{f},\lambda_{t},\lambda_{s}\right]$ as their coefficients.

Focal Loss can enforce the model to automatically focus on those hard samples, alleviating the class imbalance problem. From the perspective of classification tasks, cross entropy (CE) is a commonly used loss function. However, its mechanism of focusing on classes evenly during gradient backward makes it less effective when the distributions are imbalanced. The strategy of focal loss is reducing the value of well classified samples and increasing the value of samples that are not classified correctly. It is calculated by adding a modulating factor ${\left(1-p_{i}\right)}^{\gamma }$, also a balance coefficient. For our task, we adopt binary focal loss which can be formulated as,

(6) $$L_{focal}=-\frac{1}{N}\sum_{i}^{N}\left(\alpha_{f}g_{i}{\left(1-p_{i}\right)}^{\gamma }\log p_{i}+\left(1-\alpha_{f}\right)\left(1-g_{i}\right)p_{i}^{\gamma }\log\left(1-p_{i}\right)\right)$$

where $\alpha_{f}$ denotes the balance coefficient, *N* is the total number of pixels in the output image, $p_{i}$ refers to the $i_{th}$ pixel is predicted as a changed pixel, $g_{i}$ means the $i_{th}$ pixel in ground truth is a changed pixel.

Tversky Loss is designed following the Tversky coefficient Equation (7).

(7) $$T\left(A,B\right)=\frac{\left|A\cap B\right|}{\left|A\cap B\right|\;+\;\alpha_{t}\left|A-B\right|\;+\;\beta_{t}\left|B-A\right|}$$

where *A* and *B* represents the set of predicted positives and ground truth positives. Then |A−B| represents the set of false positive (FP), |B−A| represents the set of false negative (FN), $\alpha_{t}$ and $\beta_{t}$ controls the penalties to them, respectively. It can be observed that Tversky equals to Dice coefficients when $\alpha_{t}=\beta_{t}=0.5$, equals to Jaccard coefficients when $\alpha_{t}=\beta_{t}=1$. Basing on this coefficient, the Tversky loss is presented as

(8) $$L_{tversky}=\frac{\sum_{i=1}^{N}p_{i}y_{i}}{\sum_{i=1}^{N}p_{i}g_{i}+\alpha_{t}\sum_{i=1}^{N}p_{i}\left(1-g_{i}\right)+\beta_{t}\sum_{i=1}^{N}\left(1-p_{i}\right)g_{i}}$$

This design can adjust the recall of pixel classification in the case of loss a certain degree of accuracy when dealing with the imbalance distribution of the classification results.

SSIM Loss was originally designed to measure the structural similarity between two images. It is similar to the human visual system (HVS) and is sensitive to structural changes. Different from those methods measuring the difference between two images pixel by pixel, SSIM focuses more on the structural similarity. Let $x=\{x_{j}:j=1,\cdots ,M^{2}\}$ and $y=\{y_{j}:j=1,\cdots ,M^{2}\}$ be the pixel values of two corresponding patches with size of M×M, this loss compares the similarity of images from three dimensions: brightness, contrast and structure, presenting as:

(9) $$L_{ssim}=1-\frac{\left(2\mu_{x}\mu_{y}+C_{1}\right)\left(2\sigma_{xy}+C_{2}\right)}{\left(\mu_{x}^{2}+\mu_{y}^{2}+C_{1}\right)\left(\sigma_{x}^{2}+\sigma_{y}^{2}+C_{2}\right)}$$

where $\mu_{x}$ and $\mu_{y}$ is the average brightness of *x* and *y*, $\sigma_{x}$ and $\sigma_{y}$ is the variance of two images to present the change of contrast, $C_{1}$ and $C_{2}$ are the parameters preventing zero denominator. The structural similarity is different from the former two parts, which can be present by scalar. Therefore, the relationship between vectors composed of all pixels in two images should be normalized, that is, the covariance comparison. Then the loss function is presented as above after reduction of a fraction.

The Hybrid loss function can supervise the training process from the following aspects. Focal loss with great training stability focuses on the map pixel level detection, and considers the uneven distribution of positive and negative samples in training data, reduces the focus on the negative samples which are easy and well predicted, while increasing the focus on the positive samples. However, it ignores the information between pixels in their neighborhood. SSIM with good structure similarity comparison ability focuses on patches from the change map, and also gives higher attention to boundaries. Especially in the late training period when the focal loss trends are flat, SSIM can still ensure certain gradients to encourage the learning progression and polish the detection clearer. The introduction of Tversky loss can adjust the detection result distribution at the map level, that is, the global image level of the RCM. Combining these three loss functions, we can supervise the training process from three levels: pixels, patches, maps.

## 3. Experiments and Results

### 3.1. Datasets and Settings

Limited by manual labeling labor, the satellite image change detection datasets which can be used for deep learning are not so abundant. We implement our models on two benchmark datasets: LEBEDEV and SZTAKI.

The dataset provided by LEBEDEV [37] is one of the suitable datasets. There are two types of images in this dataset: composite images with small target offset or not, and real optical satellite images with seasonal changes, obtained by Google Earth. We apply our models on the real images, which are 11 pairs of optical images, including seven pairs of seasonal variation images of 4725×2200 pixels without additional objects and four pairs of 1900×1000 pixels with additional objects. For convenience of training, the original image sets are clipped into subset with about 16,000 image sets of real-temporal seasonal images with size of 256×256, distributed with 10000 train sets, 3000 test sets also validation sets. The results in Table 1, Table 2, Table 3 and Table 4 are performed on the 3000 test sets.

**Figure 3:**
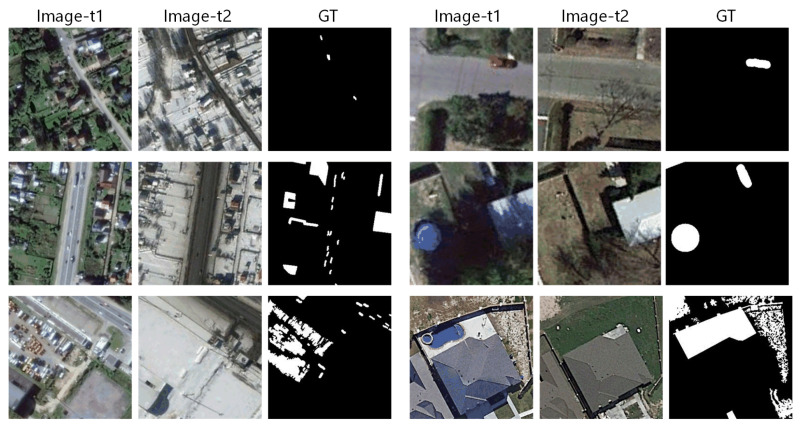
Illustration of samples from LEBEDEV. The selected bi-temporal image pairs with GT show the change including cars, buildings, surface uses, etc. The seasonal appearance difference is not considered as change. The changed area/object is marked with white pixels in GTs. It can be observed that, generally, the positive pixels are less than the negative pixels. The numbers and shapes of the changed regions are various, distributing loosely or densely, also with noises sometimes.

The spatial resolution is 3 cm to 10 cm per pixel. As shown in Figure 3, this dataset is quite challenging due to its largely seasonal differences. The manually labeled change maps only consider the appearance or disappearance of objects as the changed area, while the visual differences caused by seasonal or brightness difference are defined as the unchanged.

**Figure 4:**
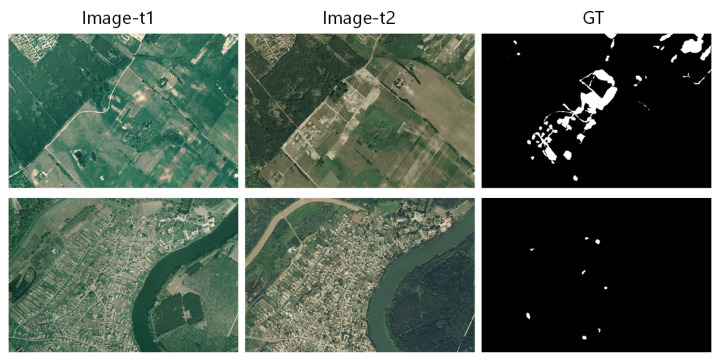
Illustrations of samples from SZTAKI. The change highlighted by white pixels contains new built up regions, building operations, planting forests, fresh plough-lands and also groundwork before building completion.

The SZTAKI AirChange Benchmark dataset contains three sets of registered optical aerial images provided by the Hungarian Institute of Geodesy cartography and Remote Sensing ($F\ddot{O}MI$). (1) SZADA consists of seven pairs of 952×640 pixels images marked manually, which were captured in 2000 and 2005, covering about 9.5 square kilometers at a resolution of 1.5 m per pixel. (2) TISZADOB consists of five pairs of images taken in 2000 and 2007 with similar resolution and size with SZADA. (3) ARCHIVE is an image obtained by $F\ddot{O}MI$ in 1984 and Google Earth in 2007, respectively. Due to the large time span and low image quality, the experiment is mainly carried out on SZADA and TISZADOB, pairs of bi-temporal pairs from each are shown in Figure 4.

To obtain the learning clips, we crop out the upper left corner with a size of 784×448 of each image as the test part. For the rest part, we apply 113×113 sliding windows clipping with overlap as the training set. The training set is augmented by rotation of $90^{\circ }$, $180^{\circ }$, $270^{\circ }$, horizontal flip and vertical flip. Following other benchmark methods [3], we choose the SZADA/1 and TISZADOB/3 from subsets as two testing sets and the rest of each as training sets, independently. The results in Table 5 are performed on these two subsets. The adopted evaluation protocols are the same as the compared methods. We implement our proposed method under PyTorch on GPU environment. During the training process, the network is optimized with a learning rate of $3\times 10^{-4}$. Based on our GPU memory, the batch size is set to 4. Furthermore, we apply auto augmentation during the training. The data loader will automatically augment the batch of images with transformations according to the random augmentation probability value, including random rotation clipping, rotation, flip and brightness, contrast, saturation changing. For loss functions, the coefficient $\left\{\lambda_{f},\lambda_{t},\lambda_{s}\right\}$ of $L_{hybrid}$ is set to {0.3,0.6,0.1}. Parameters for $L_{focal}$ are set to: $\alpha_{f}=0.75$, γ=2. For $L_{ssim}$, the sliding window size is 11, $C_{1}=0.01$ and $C_{2}=0.03$.

### 3.2. Evaluation Metrics

We evaluate the output maps and GT from four evaluation metrics: overall accuracy (OA), recall, precision and F1-score (F1). A higher recall ratio shows the less missed detection and a higher precision represents less false alarms. Thus, higher F1 shows better overall performance. These metrics can be represented as:

(10) $$OA=\frac{TP+TN}{TP+TN+FP+FN}$$

(11) $$Recall=\frac{TP}{TP+FN}$$

(12) $$Precision=\frac{TP}{TP+FP}$$

(13) $$F_{1}=\frac{2\times Recall\times Precision}{Recall+Precision}$$

where *TP*, *TN*, *FP*, *FN* denote true positive, true negative, false positive, false negative, respectively.

### 3.3. Ablation Study

**Figure 5:**
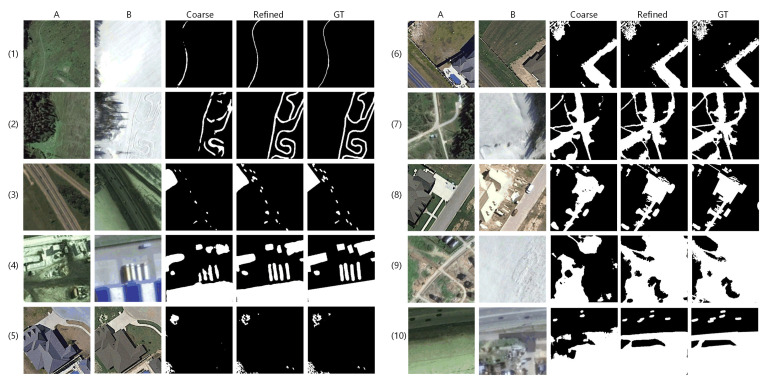
Qualitative comparison between coarse detection and refined detection.

**Figure 6:**
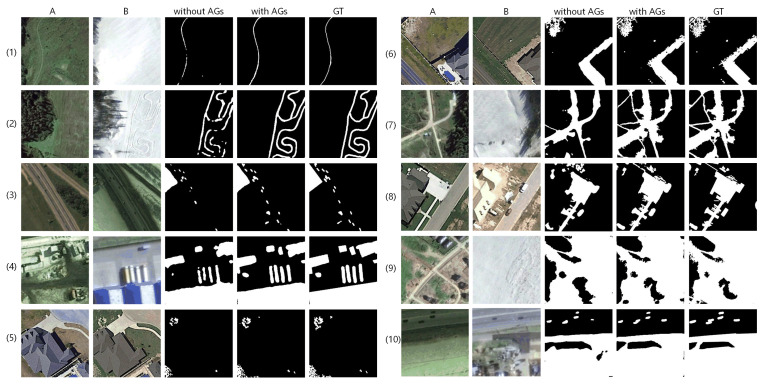
Qualitative comparison of applying AGs under coarse detection and coarse-to-fine detection.

**Figure 7:**
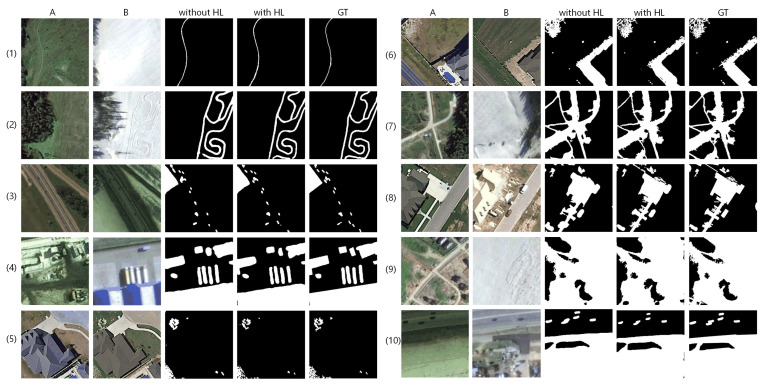
Qualitative comparison of applying hybrid loss (HL).

**Figure 8:**
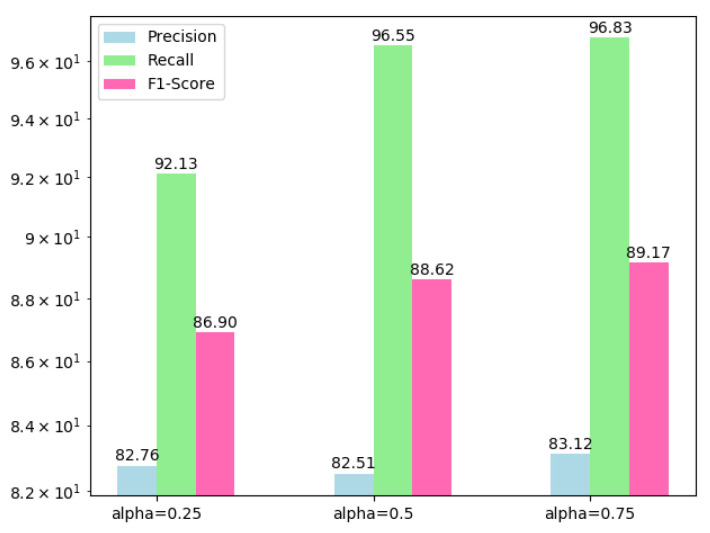
Quantitative comparison of $\alpha_{f}$ for focal loss.

In this part, we will discuss the effectiveness of coarse-to-fine detection framework, AGs, hybrid loss, by implementing ablation networks on LEBEDEV with quantitative and qualitative comparison, respectively. For quality discussion, we select 10 sets of challenging samples with obvious seasonal differences, showing in Figure 5, Figure 6 and Figure 7. Quantitative discussion revolves around the precision, recall and F1 scores in Table 1, Table 2 and Table 3 and Figure 8. The changed areas include: slender pathway change in wide background; multiple objects change in complex sizes and shapes; dense and piece like irregular shaped change instead of compact changed area; change disturbed by seasonal differences of plants, also the largely difference of brightness etc.; complex change situation with large area. The proportion of positive and negative samples in each row of each group increases gradually.

#### 3.3.1. Effectiveness of Coarse-to-Fine Detection Framework

To observe and verify the effectiveness of our coarse-to-fine framework, we compare the change maps obtained by coarse detection and refined detection. The quantitative comparison showing in Table 1 indicates that the refined detection can obviously improve all evaluation scores. Though the score improvements are not significant, the qualitative comparison showing in Figure 5 illustrates that refined detection leads to clearer boundaries which are closer to GTs. These visual enhancements at the boundary may not be sensitive to quantitative measurements.

It can be observed that, through the quality comparison illustrated in Figure 5, the coarse detection framework can locate most of the changed areas with the help of AGs, but there are obvious interior parts vanishing, inaccurate boundary and imperfect interior compactness. In addition, when hybrid loss is used without refined learning, few of missed detection appear in extreme small areas. After the refined detection, the above mentioned problems have been mainly solved, including visually GT-closer boundary and rich details accurate description on all showing samples, especially on samples (5), (6) and (8). Furthermore, the missing parts are complemented on samples (1), (2), (7), (9) and (10). These comparisons clearly show the effectiveness of the refined detection framework.

#### 3.3.2. Effectiveness of Attention Gates

The quantitative comparison is shown in Table 2. It can be observed that the introduction of AGs significantly enhances the recall (3.71%) with a slight reduction of 0.4% in precision, so F1 score improves by 1.5%. Such quantitative comparison shows that AGs can help improve recall and F1 score obviously on the basis of slight fluctuation in precision. This indicates that AGs allow the network to have stronger ability of detecting more changed areas. It benefits from the guidance from deeper features that capture more contextual information.

Qualitative comparison in Figure 6 can strongly prove the effectiveness of AGs. It can be observed that the change maps generated by the BA^2^Net cutting off AGs, have accurate shape and visually closer boundary to GTs but also obvious false detection. The falseness is mainly about false negative detection (samples (3)–(8)) and a few of false positive detection (samples (1), (8)). The reason for this phenomenon may be that the network without AGs gives almost equally attention to low-level and high-level features, which leads to obvious false detection in several samples, such as extremely unbalanced positive and negative patches and multiple changed areas patches. Change maps obtained under the guidance of AGs involving more high level features are more semantically accurate and are able to locate more changed areas accurately.

#### 3.3.3. Effectiveness of Hybrid Loss

To prove the effectiveness of hybrid loss, we perform ablation study on losses with the same experimental setup (batch size = 4 and epoch is set to at least 60, the training process will stop when F1 score does not improve for 20 continuous epochs). As shown in Table 3, by equipping hybrid loss onto BA^2^Net, the precision and F1 scores increase largely. Hybrid loss can make an increase of 5.26% on precision while just a reduction of less than 1%. These show that hybrid loss can significantly improve the detection precision while losing a few detection rates, also help improve the best F1 score of 91.36%.

The qualitative comparison is illustrated in Figure 7.

It can be observed that without using hybrid loss, BA^2^Net can mainly detect and describe the changed areas accurately. However, there are a few missing detection for extreme small changed areas (sample (8)), and the boundary description of the densely gathered piece like areas (sample (5), (6)) is not clear and explicit enough. Under supervision of hybrid loss, the elongated changed area in the upper part of change map (8) is correctly detected; the dense boundary in the map (5) and (6) is clearer and distinguishable; the changed area boundary of all shown maps is closer to GTs based on original detection.

To discuss sub-items of hybrid loss, we train BA^2^Net under weighted combinations of these sub-items and quantitatively compare them in Table 3. Due to the extremely slow convergence of training with Tversky or SSIM loss individually, we neglect the comparison of these two situations. Compared with BCE loss, by introducing the modulating factor ${\left(1-p_{i}\right)}^{\gamma }$, focal loss concentrates on the foreground, which makes its all scores increase slightly compared with BCE. It reaches 83.12%, 96.83% (top), 89.17% in precision, recall and F1. However, it is obvious that the recall scores of the two loss functions based on cross entropy are much higher than (about 13% difference) precision scores, which indicates that the learning on unchanged area pixels may be ignored while emphasizing the changed pixels. After involving Tversky loss, the difference between precision and recall is reduced to 8.51% (precision increased by 3.12% and recall reduced by 2.08%) and F1 score increased slightly. With the introduction of SSIM loss, all scores improve on previous a basis, among which the precision and F1 reach the highest values in our experiments.

To select the value of $\alpha_{f}$ from focal loss, we implement the BA^2^Net when $\alpha_{f}=\left\{0.25,0.5,0.75\right\}$ while γ=2 and visualize the quantitative comparison in Figure 8. Based on this comparison, we set the $\alpha_{f}=0.75$ to maintain an effective performance.

To sum up, sufficient ablation experiments have respectively proved the effectiveness of designs of our BA^2^Net. The introduction of coarse-to-fine detection framework can boost network performance in several aspects, including more correct detection and more accurate boundary labeling. The application of AGs greatly improves the detection rate, which can be observed in terms of quality and quantity. Hybrid loss is also obvious for quantitative enhancement, and allows our model to work well for some challenging to identify boundaries.

### 3.4. Result Comparisons

To verify the effectiveness and superiority of our proposed BA^2^Net, we compare it with some SOTA methods, introducing briefly as:

1. CD-Net [35] is a pixel-wise change detection net, which is constructed on the structure of a typical Siamese network with contraction blocks and expansion blocks. The change map is generated by a Softmax layer.
2. DSCN [22] trains AlexNets sharing parameters and cutting off the pooling layers as the streams of Siamese network. By discarding pooling operations, it keeps the respective field and feature maps dimensions.
3. FC-EF [3] refers to fully convolutional early fusion. It stacks an image pair as one input and feeds it into a standard U-Net. This network is structurally simple and effective.
4. FC-Siam-conc [3] processes each image by the encoder part of U-Net, separately. Furthermore, then concatenates each feature map pair in encoding connecting into the decoder part by skip connections.
5. FC-Siam-diff [3] is a network similar to FC-Siam-conc. The different point is its skip connections link with the difference of encoding feature map pairs, instead of concatenating directly.
6. FCN-pp [32] is an FCN applied with pyramid pooling which can capture a wider receptive field and overcome the drawbacks of global pooling.
7. DSMS-FCN [5] proposes a unit that is able to extract multiscale features in the same layer. Based on the proposed unit, deep Siamese multiscale fully convolutional network is designed for supervised change detection. The structure is similar to the FC-Siam-diff.
8. UNet++MSOF [2] is a 2-channel network introducing multiple dense intermediate nodes and skip connections into standard U-Net. The advantage of this structure is that it can learn from multiple scale feature maps more automatically. While using deep supervised strategy by obtaining four output maps supervised by multiple side-outputs fusion (MSOF) loss. It has excellent performance on LEBEDEV, but for the boundary accuracy and multiple small objects, it still can be improved.
9. IFN [1] utilizes a feature extraction network with shared parameters to encode the original images, which is similar to the FC-Siam-conc. In the decoding process, it implements discrimination learning on the difference between the features of each layer in the former streams. Different from the FC-Siam-conc, it applies the deep supervision strategy to enhance the performance on the boundary integrity and internal compactness. However, it still misses a few objects when images contain multiple objects.

For quantitative comparisons, the evaluation metrics were calculated and summarized as shown in Table 4 and Table 5, on LEBEDEV and SZTAKI, respectively. The best scores are highlighted with bold and red, while green and blue indicate the second best and the third best, respectively.

As can be observed from the Table 4, CD-Net and DSCN scores are the lowest and far lower than other methods. CD-Net operates decoding directly on the features obtained by encoding layers to generate original sized change maps, while DSCN abandons the pooling operations in the common process of convolutional encoding to maintain the respect fields and the feature maps sizes. These simple strategies without design of cross-scales features learning result in a certain amount of error accumulation, which leads to their low scores in all measurement metrics. Besides these two networks, all methods are designed to build on the encoder-decoder structure with cross-scale designs. By using skip connections with different fusion strategies, FC-EF, FC-Siam-conc and FC-Siam-diff achieve higher scores. Among them, FC-EF using the early fusion strategy boosts F1 scores by about 10% than the previous two networks. On the basis of that, by applying late fusion strategy, FC-Siam-conc and FC-Siam-diff improve their F1 scores by about another 5%, achieving at around 83%. In order to further utilize the multiscale features, FCN-PP introduces pyramid pooling on the FCN framework. Compared with FC-EF, which has a similar framework, FCN-PP improves the F1 score by about 3% but is still lower than FC-Siam-conc and FC-Siam-diff. Instead of conventional convolution units, DSMS-FCN designs a multiscale convolution unit to utilize features in multiple scales. Compared to its backbone framework FC-Siam-diff, it enhances all evaluation scores obviously.

The top three scores are concentrated in UNet++MSOF, IFN and our BA^2^Net which further enhance the evaluation scores compared to the previous methods. For the multi-scale issue, UNet++MSOF adopts UNet++ as the detection model. This fully and densely design of automatically learning strategy can take fully use of multiple features at all scales. At the same time, through the combination of four shallow outputs supervised by its multiple fusion loss, UNet++MSOF reaches superior results than previous methods. Based on the framework of FC-Siam-conc, IFN introduces spatial and channel attentions and gradually carries out supervised fusion in the process of decoding. IFN achieves the current highest precision (94.96%) due to its design for sufficient depth and parameters on multiple scales.

These two networks have rich designs for multiple scale features, which ensures good precision. However, they pay nearly even attention to multiple scales and weakly to help the detection rate of changed areas. Our proposed BA^2^Net reaches the current highest recall, under the premise of the third precision, which is attributed to the introduction of attention mechanism guided by deeper features into our network. Such a mechanism allows a higher ability to locate more changed areas under the guidance of more semantic context information.

**Figure 9:**
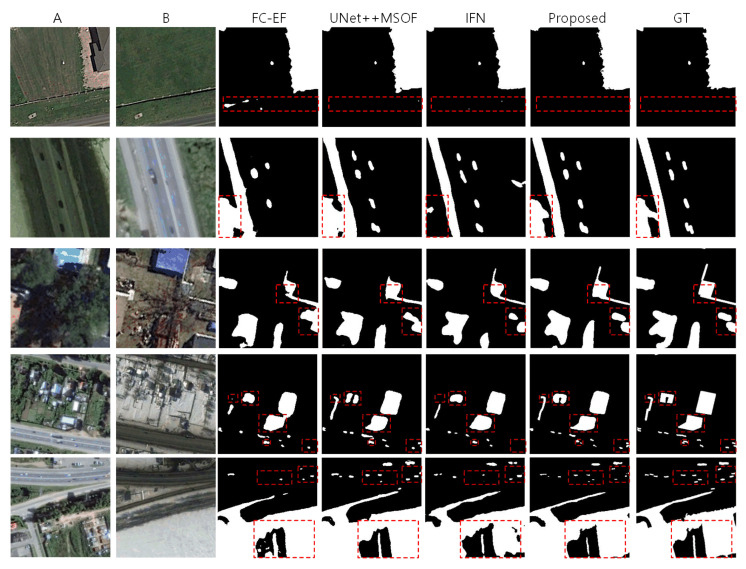
Qualitative comparison between our proposed BA^2^Net with other benchmark SOTA methods. Five rows are five sets of ‘$I_{t1}$, $I_{t2}$, FC-EF, UNet++MSOF, IFN, BA^2^Net’, arranging the samples are becoming more and more challenging. The bi-temporal images in the first two rows are samples with multiple small regions changing and enclosing walls, land surface changes. These samples are not quite challenging pairs. Both SOTA and proposed maps perform well, but there are missing detection and false alarms in the SOTA maps, highlighted by the red boxes, while our model labeling correctly. The third sample is more challenging due to the region covered by seasonal appearance changes of trees. Our model is more accurate when representing the changes in the above challenging regions. The fourth and fifth samples contain largely seasonal appearance changes, multiple areas with various shapes. In the highlighted boxes, our results outperform the SOTA maps from correctness of localization and the accuracy of boundary depicting.

To qualitatively compare with other SOTA methods, we select top-2 methods (UNet++MSOF, IFN) other than our proposed method and a classic framework (FC-EF) as the quality comparison. As illustrated in Figure 9, the proposed BA^2^Net is obviously superior to other methods in quality.

As the showing samples shown in Figure 9, five sets illustrate the effectiveness of our model with positive areas from less to more. In the first set, there are certain false positive detection for FC-EF, and minimal false positive for UNet++MSOF and IFN. Furthermore, for the upper right region of change with jagged detail, other comparing methods are weaker in describing edges than our model. The second set contains multiple variation changed regions, FC-EF and IFN appear obvious regional missed detection and false positive. UNet++MSOF is more accurate, but the shape errors in the lower left corner are obvious. The third group has various changed regions with smooth shapes. On the basis of basically locating the variation areas, our model is superior to other methods in the accurate description of shapes. The fourth group is similar to the third group but more challenging, with smaller, more shapes-rich regions. FC-EF and IFN miss detection on multiple small pieces of changed regions, and the shape description is less accurate. UNet++MSOF has no obvious mistake locating in detection, but the edges are not exact enough. Comparatively, our model performs particularly well on this type of data, being able to basically locate all the various regions and to describe shapes exactly. The variation areas in the fifth set are characterized by multiscale and multi-shape. It can be observed from the highlighted boxes that our model is obviously superior to other methods.

On the dataset of SZTAKI quantitative summarization Table 5, the subsets SZADA/1 and TISZADOB/3 are treated as two test-sets separately. It can be observed that the top three scores are not concentrated in a few methods, but are scattered in various methods. At the same time, though both subsets belong to one dataset, except for the proposed model, none of the other comparing methods can simultaneously be ranked in the top three on F1 scores.

The two methods with weak performance in the LEBEDEV, CD-Net and DSCN are also relatively weak in SZADA/1, while the DSCN reaches a third precision on TISZADOB/3 and a good F1 score. The three models (FC-EF, FC-Siam-conc, FC-Siam-diff) that perform well on LEBEDEV work still well on these subsets, with FC-Siam-diff reaching SZADA/1’s second best F1 score and FC-EF ranking first on TISZADOB/3. FCN-PP, which introduces pyramid pooling, is similar to FC-EF on SZADA/1 and surpasses some other complex frameworks. Although DSMS-FCN reaches the top F1 score and precision on SZADA/1, it is the lowest F1 score and recall on TISZADOB/3. UNet++MSOF, which has an advantage in precision, has the highest precision on TISZADOB/3. IFN, on the other hand, is relatively weak on both these subsets. Our model ranks the third and the second best F1 scores and reaches the highest recall on these subsets.

In general, there is hardly a method with absolute superior performance over the SZTAKI. By utilizing more multi-scale features, DSMS-FCN and UNet++MSOF obtain the highest precision in these two subsets, respectively. However, neither of them can guarantee excellent effectiveness on both subsets, especially DSMS-FCN’s performance on TISZADOB/3 ranks much lower than on SZADA/1. Benefit from high-level features guided attention mechanism, our model shows a stable and excellent performance in recall, and is the highest model on both datasets. In addition, it ranks second and third in F1-score, respectively, and the robustness of the proposed method is obviously superior to the compared methods. In terms of F1 scores, the models with the highest scores have no obvious commonality except that both are based on the FCN framework. Though proposed model have no obvious advantage in precision may due to the limited training data, it still shows stable and promising recall and competitive F1 score.

**Figure 10:**
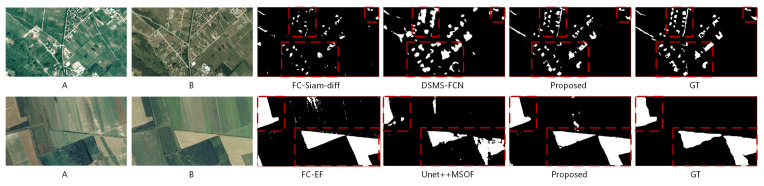
Illustration of our RCMs on SZTAKI with SOTA methods. Two rows show in order by SZADA/1 and TISZADOB/3, A and B mean the bi-tempral image pair. The comparing methods are FC-Siam-diff, DSMN-FCN, FC-EF and UNet++MSOF. In the first row, our RCM shows more accurate multiple winding shaped small areas describing, highlighted by the boxes. In both rows, our BA^2^Net represents GT-closer edges of the large range of changed areas.

Meanwhile, we illustrate qualitative comparison with the top three quantitative methods in Figure 10 on SZADA/1 and TISZADOB/3.

In the bi-temporal image pair from SZADA/1, it contains a large number of multiple small objects with sorts of appearances. Our model is more accurate than the other top three ranking methods in expressing the shape form of the changed regions, especially the region with more winding boundaries. However, there are some false positive dots in the broad background region. In the pair has large areas of change with smooth edges, the TISZADOB/3, our model performs well. It can accurately locate the large changed areas and clearly represent edges.

To sum up, abundant comparative experiments on LEBEDEV and SZTAKI prove the effectiveness of our BA^2^Net. From the perspective of qualitative analysis, our model shows the boundary delineation ability superior than other methods in both datasets, which can represent boundary clearer and closer to GTs. Furthermore, our model can detect and locate multiple changed areas in excellent detection rate. In quantitative analysis, the promising recall on both datasets also illustrate the detection capability of our model. However, the visually closer boundary may not be sensitive to precision, so there is no obvious advantage in precision. In combination with the above, there are still advantages in F1 scores of our model.

## 4. Discussion

Either the traditional change detection methods, or the deep learning based following the strategies of traditional methods, such methods have a certain amount of error accumulation in the process of extracting features, comparing features, setting thresholds or clustering. Therefore, the image-to-image is currently considered as a superior framework. Thus, it demands to develop an end-to-end network base on this framework to generally learn the changes of bi-temporal images.

Our proposed network was validated on two benchmark datasets. By qualitative and quantitative comparing, we can observe that our model is superior to other models in that the image contains multiple changed areas (from very small areas to a wide range of areas, from compactness within regular shapes to piece like various shapes), especially when the changed area consists of densely gathered pieces which are challenging to distinguish their boundaries. That is, it can locate each changed area correctly, and it can clearly and accurately delineate the changed area boundary. This is one of the significant important measures for satellite image change detection. This superiority may benefit from the introduction of the attention mechanism and refined detection collaborative with a hybrid loss. By using them, the BA^2^Net can focus on the positive samples in the case of uneven positive and negative samples under the attention gates and modulating factors, while ensuring the consideration to the negative samples to ensure sufficient gradient-driven training at a later stage, and a comprehensive method of comparing structural similarity with the distribution of map classification results. In addition, our model is designed following end-to-end, and the good flexibility of the network ensures its applicability, whether it extends from image pairs to multiple image inputs or changes from visible images to multimodal data with only to modify the pre-processing.

The proposed network has some limitations. First, although attention mechanism is introduced to increase the focus in the foreground when there is less positive GT pixels, the problem is that it does not adequately select between high-level and low-level features, which results in an unstable attention mechanism when some of positive and negative samples are evenly distributed. The dense prediction strategy along with multiple outputs supervision performs well in stability when processing changes in multiple scales, thus how to combine this strategy with the multiscale guided attention can be studied. Second, on the SZTAKI dataset, our method is not significantly superior to others. This probably be limited by limited training data. The emergence of generative learning, weak supervised learning and knowledge distillation in recent years probably can solve these problems. How to introduce these mechanisms into change detection is one of the next research directions.

## 5. Conclusions

In this paper, we propose a BA^2^Net with a hybrid loss to detect changes in VHR satellite images. The existing methods have achieved good results by introducing the encoding-decoding models utilizing semantic information, but there are still some weaknesses when there are multiple small changed areas, such as miss detection all the areas, false detected the changes of areas and inaccurate description of areas boundary. In view of this, we propose to use a coarse-to-fine framework to detect the change of image pairs, including a higher-level features guided coarse detection, and a refined residual detection. At the same time, we use hybrid loss to supervise the training from pixel, patch, and map levels, which ensures the stability of training and adapt the case of imbalance distribution of positive and negative samples. Experiments on two benchmark datasets show that our model is superior to other methods.

## Figures and Tables

**Table 1:** Ablation study on coarse-to-fine detection framework.

BA^2^Net	Precision	Recall	F1
Coarse Detection	85.09	93.79	88.99
Refined Detection	88.12	95.28	91.36

**Table 2:** Ablation study on attention gates of BA^2^Net.

BA^2^Net	Precision	Recall	F1
without AGs	88.52	91.57	89.86
with AGs	88.12	95.28	91.36

**Table 3:** Ablation study on hybrid loss of BA^2^Net.

Losses	Precision	Recall	F1
BCE	82.86	96.12	88.39
Focal	83.12	96.83	89.17
Focal + Tversky	86.24	94.75	90.13
Focal + Tversky + SSIM	88.12	95.28	91.36

**Table 4:** Quantitative results on LEBEDEV of BA^2^Net and other SOTA methods.

Methods	Precision(%)	Recall(%)	F1-Score(%)	OA(%)
CD-Net [35]	73.95	67.97	68.82	91.05
DSCN [22]	79.18	55.74	65.07	94.04
FC-EF [3]	81.56	76.13	77.11	94.13
FC-Siam-conc [3]	84.41	82.50	82.50	95.72
FC-Siam-diff [3]	85.78	83.64	83.73	95.75
FCN-PP [32]	82.64	80.60	80.47	95.36
DSMS-FCN [5]	88.60	84.85	86.61	96.20
UNet++ MSOF [2]	89.54	87.11	87.56	96.73
IFN [1]	**94.96**	86.08	90.30	97.71
BA^2^Net	88.12	**95.28**	**91.36**	**98.94**

**Table 5:** Quantitative results on SZTAKI of BA^2^Net and other SOTA methods.

Methods	SZADA/1			TISZADOB/3		
	Precision(%)	Recall(%)	F1-Score(%)	Precision(%)	Recall(%)	F1-Score(%)
CD-Net [35]	40.35	41.81	40.42	82.37	87.28	79.70
DSCN [22]	41.20	57.40	47.90	88.30	85.10	86.70
FC-EF [3]	43.57	62.65	51.40	90.28	96.74	**93.40**
FC-Siam-conc [3]	40.93	65.61	50.41	72.07	96.87	82.65
FC-Siam-diff [3]	41.38	72.38	52.66	69.51	88.29	77.78
FCN-PP [32]	42.97	69.39	51.91	85.11	89.89	86.57
DSMS-FCN [5]	**72.31**	44.77	**55.30**	80.09	53.77	62.66
UNet++MSOF [2]	41.67	69.54	51.01	**93.61**	85.80	87.56
IFN [1]	42.55	53.01	45.92	81.58	89.86	84.94
BA^2^Net	40.53	**79.82**	52.33	87.19	**97.13**	91.61

## Abbreviations

The following abbreviations are used in this manuscript:

SOTA	state of the art
BA^2^Net	boundary-aware attentive network
CM	change map
CCM	coarse change map
RCM	refined change map
VHR	very high resolution
SAR	synthetic aperture radar
CNN	convolutional neural network
DBN	deep brief network
SDAE	sparse de-noising autoEncoder
FCN	fully convolutional network
BN	batch normalize
ReLU	rectified linear unit
AG	attention gate
GT	ground truth
TP	true positive
TN	true negative
FP	false positive
FN	false negative
OA	overall accuracy
CE	cross entropy
BCE	binary cross entropy
SSIM	structural similarity
HVS	human visual system
CD-Net	change detection network
FC-EF	fully convolutional early fusion
FC-Siam-conc	fully convolutional Siamese concatenation
FC-Siam-diff	fully convolutional Siamese difference
FCN-PP	fully convolutional network with pyramid pooling
DSCN	deep Siamese convolutional network
DSMS	deep Siamese multiScale
MSOF	multiple side-outputs fusion
IFN	image fusion network
